# A machine learning approach to predict river hydrographs for water quality sampling

**DOI:** 10.1007/s10661-026-15786-0

**Published:** 2026-08-17

**Authors:** Eloise Wilson, Melanie Shaw, Frederick Bennett, Zoe Bainbridge, Stephen Wallace, Ryan Turner

**Affiliations:** 1 Reef Catchments Science Partnership, School of the Environment, The University of Queensland, Brisbane, Queensland 4072 Australia; 2 Catchment Water Quality Alliance, Brisbane, Queensland Australia; 3 Water Quality Modelling, Queensland Department of the Environment, Tourism, Science and Innovation, Brisbane, Queensland 4102 Australia; 4 Catchment to Reef Research Group, TropWATER, James Cook University, Townsville, Queensland 4811 Australia; 5 Water Quality and Investigations, Queensland Department of the Environment, Tourism, Science and Innovation, Brisbane, Queensland 4102 Australia

**Keywords:** Adaptive sampling, Decision support tools, Event-based sampling, Pollutant load estimation, Probabilistic regression, River height forecasting

## Abstract

Hydrology and water quality are innately linked, as flow dynamics control the transport of pollutants within river systems. Consequently, the timing of sample collection in water quality monitoring programs strongly influences the accuracy of estimated pollutant loads. To minimise error, concentrations should be sampled across the hydrograph, capturing the rising limb, peak, and falling limb, to reflect the dynamic nature of pollutant transport. However, in river systems with variable flow regimes, predicting the timing and duration of events is challenging. Monitoring programs often resort to oversampling to ensure that critical periods are represented, but this approach increases effort and cost. In this study, we apply probabilistic gradient boosting decision tree regression (CatBoost) to forecast river height in a tropical, fast-response catchment characterised by high-flow variability, using hourly rainfall, discharge, and river height data collected over a 15-year period. Model performance was evaluated across forecast horizons ranging from 1 to 48 h. The models reproduced hydrograph magnitude, shape, and timing with high accuracy at short horizons (1–12 h), while forecast confidence and accuracy declined progressively at longer horizons (24–48 h). Forecast performance also varied across flow regimes: low flows were predicted accurately across all horizons, moderate flows reliably up to the 24-h horizon, and high flows up to the 12-h horizon. Predictive skill declined for extreme events; however, forecasts remained operationally valuable up to the 12-h horizon. These findings highlight the potential for short-term forecasts to support adaptive, resource-efficient sampling programs and reduce reliance on oversampling while maintaining pollutant load accuracy.

## Introduction

Machine learning offers a promising approach to improving river water quality sampling regimes in an adaptive way (Varadharajan et al., 2022). By drawing on long-term hydrological and water quality records, machine learning models have proven effective for predicting river level dynamics (Hu et al., 2020; Rathnayake et al., 2023; Shiri et al., 2023; Xu et al., 2020). Machine learning has been applied to hydrologic forecasting across a range of conditions and forecast horizons. For example, Almikaeel et al. (2025) demonstrated its potential to accurately predict river height, with a particular emphasis on extreme hydrological events. Their model used 36 h of rainfall, discharge, and river height observations to predict the subsequent 12 h of river height. Similarly, Kashem et al. (2024) evaluated forecasting accuracy across horizons ranging from 1 day to 3 weeks, finding that forecast skill declined as horizon length increased, with 1-day-ahead predictions yielding the most accurate results. Machine learning models have also been applied in small, fast-responding catchments for near real-time prediction. In their study, Luppichini et al. (2024) used a long short-term memory (LSTM) encoder–decoder network to forecast river height at 15-min intervals, with 1- to 6-h horizons. The model incorporated upstream rainfall and river height inputs from multiple sensors across mountainous catchments in Tuscany, Italy, and highlighted the ability of machine learning models to track hydrograph dynamics in near real time, particularly during peak flows. Although these studies demonstrate the potential of machine learning in hydrological forecasting, to our knowledge it has not yet been applied directly to water quality monitoring programs designed to calculate pollutant loads.

Current approaches to pollutant load sampling often rely on site-specific trigger levels or manual judgments about how river height is likely to change during rainfall events. This typically involves anticipating the timing, shape, and magnitude of an event using observed conditions (e.g. forecast rainfall, antecedent conditions, and upstream flows). However, if sampling is mistimed or key moments such as the event peak are missed, substantial error can be introduced, resulting in pollutant load estimates that do not reflect actual conditions (Lewis et al., 2007; Marsh & Waters, 2009; Thomson et al., 2012). Machine learning models can be trained on long-term, high-resolution (e.g. hourly or sub-hourly) environmental datasets to predict the duration, shape, and peak timing of hydrographs (Luppichini et al., 2024). These predictions may assist more strategically timed event-based sampling, reducing uncertainty and improving the accuracy of pollutant load estimates.

Here, we explore the use of gradient boosting decision trees (CatBoost) to forecast river height across multiple forecast horizons. This study aims to assess the feasibility of machine learning model application to inform and or improve water quality sampling regimes, with the goal of minimising errors in pollutant load estimates and improving resource efficiency. Our objective is to model the shape and timing of hydrographs (river height) across a range of flow regimes to inform adaptive sampling for water quality monitoring. This extends previous work, which has either focused on peak flow or extreme value prediction (Almikaeel et al., 2025; Dong et al., 2021; Lin et al., 2023; Luppichini et al., 2022, p. 202; Zehra, 2020), or applied machine learning methods to forecast full flood hydrographs, including rising and falling limbs, for streamflow (discharge) prediction and hydrological forecasting (Tayfur et al., 2018; Yaseen et al., 2018). We test a machine learning–based approach by training separate CatBoost models for each forecast horizon using observed rainfall, discharge, and river height inputs to evaluate the capacity of a scalable forecasting model to support near real-time sampling decisions, using a tropical river catchment as a case study.

## Background

### Challenges in water quality sampling for pollutant load estimates

**Fig. 1:**
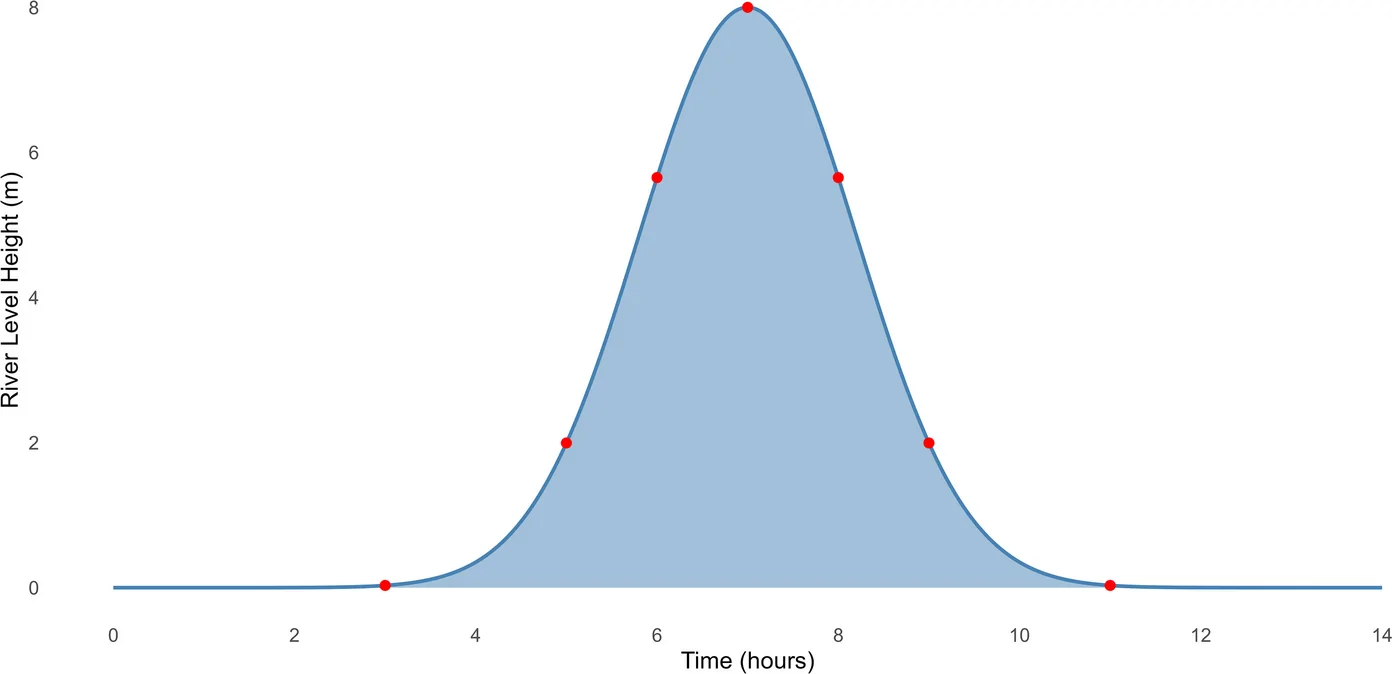
Example hydrograph representing a high-flow event at a fine-scale site, with sample points distributed to capture two to three on the rising limb, one at the peak, and two to three on the falling limb (adapted from Lewis et al., 2007)

Reliable pollutant load estimation requires the collection of water samples to adequately capture continuously changing flow and concentration conditions within a waterway. Sampling regimes must consider the number of samples collected and the timing of their collection to meet this (Thomson et al., 2012). Inadequate sampling, whether in terms of data volume, frequency, or coverage, introduces uncertainty into pollutant load estimates (Letcher et al., 2002; Marsh & Waters, 2009). Studies across tropical and sub-tropical fine-scale (e.g. drains or small waterways) and catchment-scale sites have evaluated the relationship between sample coverage and the error associated with pollutant load estimates. One example of water quality sampling during extreme flow events in a tropical fine-scale site found that the most reliable pollutant load estimates were obtained when samples were collected evenly across the hydrograph, specifically two to three samples on the rise, one at the peak, and two to three on the fall (Fig. 1; Lewis et al., 2007). Sampling of the rising limb and the peak of an event is particularly important because these phases often coincide with the first flush of sediment and nutrients, when concentrations are highest and loads increase most rapidly (Davis et al., 2016; Obermann et al., 2007). In another study, Marsh and Waters (2009) evaluated how pollutant load estimates vary under different sampling scenarios across tropical and sub-tropical catchments. They showed that estimates based on fewer than eight samples rarely fell within 50% of the true mean, while the most accurate results were obtained when more than eight samples were distributed across both the rising and falling limbs of the hydrograph. Thomson et al. (2012) evaluated how different sampling strategies, including frequency and hydrograph coverage, affect load estimation error with the aim of providing a framework to guide practitioners in selecting suitable methods for different sampling scenarios in northern Australia. This study found that the greatest error in load estimations occurred when sampling was not undertaken on the fall of the hydrograph and recommended that samples, whenever practical, be collected across both the rise and falling limbs. They also showed that mean root mean square error (RMSE) values and inter-event variability were lowest when samples covered both limbs of the hydrograph and highest when neither limb was sampled. These studies demonstrate the potential for error when sample collections are mistimed and highlight the importance of capturing key periods within the event hydrograph to ensure that pollutant loads are accurately estimated.

In practice, it can be challenging to time event sampling to ensure appropriate coverage, and continuous sampling is rarely feasible. Events may occur outside working hours, and automated samplers are constrained by the number of bottles they can store. Whether the collection is manual or automated, these limitations mean that only a finite number of samples can be collected during a given event. Restrictions also apply to analyte holding times, defined as the period between when a sample is collected and when the analytical process begins. For pollutant load estimation in river water quality monitoring, the common limiting analytes are nutrient species, which must be retrieved from automated samplers within 24 h to allow subsequent freezing and longer-term storage (Standards Australia, 1998a, b). This requirement constrains the timeframe for triggering based on predicted flow and adds an additional layer of complexity to event-based sampling. As a result, the ability to predict the shape and timing of a hydrograph would enable practitioners to target sample collection at critical periods, reduce the potential for error in load estimation, and allocate resources more effectively to ensure adequate hydrograph coverage.

## Materials and methods

### Study area

**Fig. 2:**
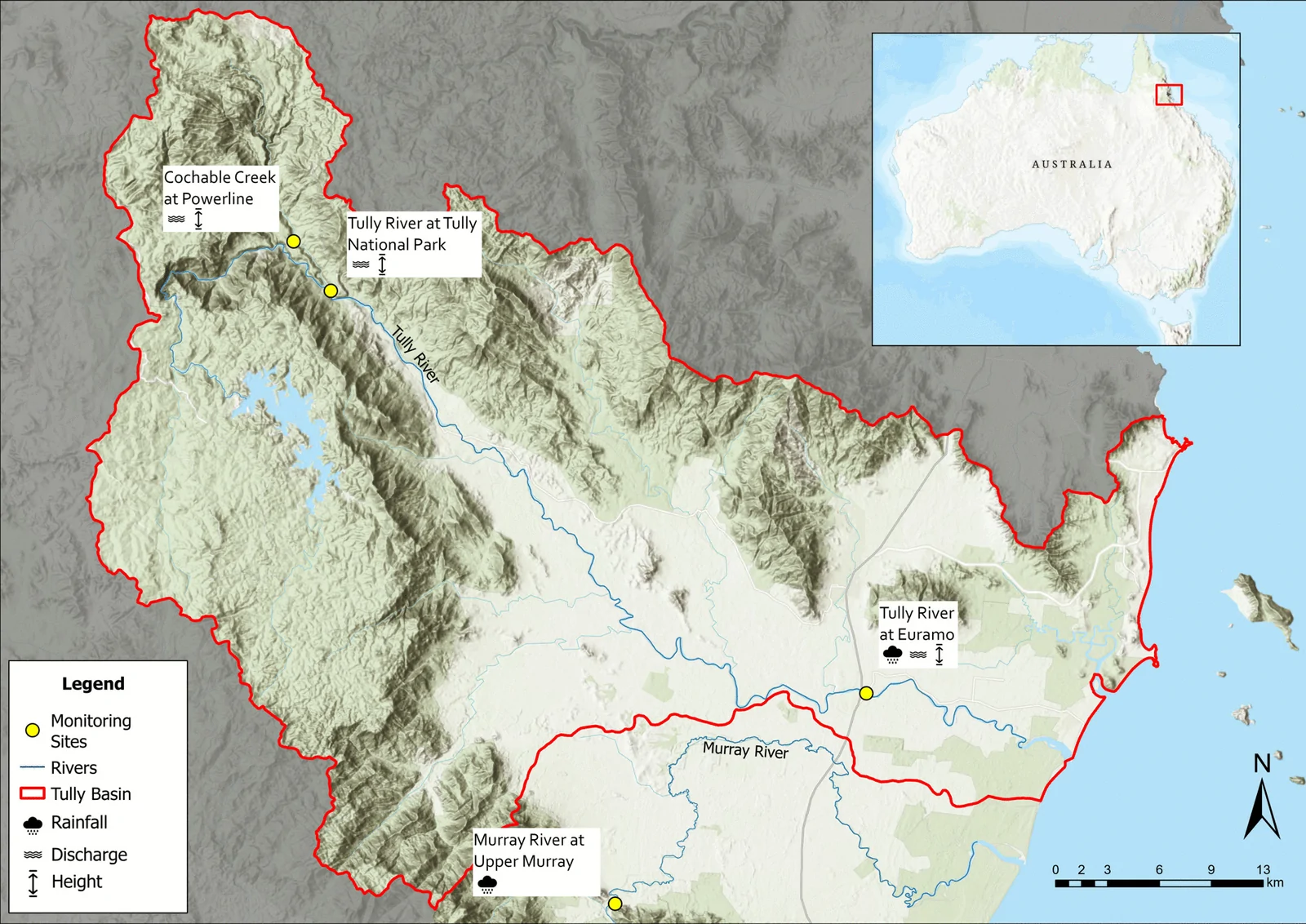
Map of the Tully catchment in north Queensland, Australia, indicating the monitoring sites (yellow circles) that provided predictor variables for the forecasting models

The Tully catchment, located in the Queensland Wet Tropics bioregion, covers an area of approximately 1683 km^2^ (Karim & Wallace, 2008; Fig. 2). Its topography ranges from steep, rainforest-covered mountains in the upper catchment to a broad, low-relief coastal floodplain. The floodplain is predominantly used for agriculture, although patches of remnant forest persist along parts of the coastal fringe (Armour et al., 2009; Pearson et al., 2011). The regional climate is tropical, with annual rainfall typically between 2000 and 4000 mm (Peel et al., 2007). Around 80% of the annual rainfall occurs during the wet season from December to May (Australian Bureau of Meteorology, 1960–2010). Streamflow records from the Tully River at Euramo gauge (113006A) over a 30-year period indicate a mean annual discharge of 1.1 million megalitres (ML), with interannual totals varying from 1.0 to 6.0 million ML. The Tully has the highest discharge per unit area of any Australian catchment (Furnas, 2003; McCloskey et al., 2021).

Our study site, the Tully River, is a small coastal river with a high-energy hydrological regime, in which most pollutants are transported to the adjacent coast (McCloskey et al., 2021). Beyond the coastline, the river water flows directly into the Great Barrier Reef lagoon and is therefore closely monitored for its contribution of pollutants, including pesticides, nutrient species, and suspended sediments. The floodplains are typically inundated three to four times per year, with wetland connectivity varying depending on the magnitude of each flood. During major events, water from the Tully and Murray rivers often converges, creating a single, extensive inundated area. Flooding primarily occurs during the wet season, with the largest flows usually observed in January, when several cubic kilometres of water can spread across the floodplain (Karim & Wallace, 2008; McJannet et al., 2012; Wallace et al., 2009).

### Model selection

Data-driven machine learning regression models have become central to streamflow prediction, offering robust alternatives to traditional hydrological models across a wide range of scenarios (Ahmed et al., 2022; Luppichini et al., 2024; Nguyen et al., 2015; Phan & Nguyen, 2020; Tien Bui et al., 2020). Machine learning regression models such as Support Vector Regression (SVR) (Sharma and Goel 2024), Random Forest (RF) (Kedam et al., 2024), Extreme Learning Machine (ELM) (Adnan et al., 2023), Convolutional Neural Network (CNN), Long Short-Term Memory (LSTM) (Kratzert et al., 2018; Workneh & Jha, 2025), and ensemble methods (e.g. Gradient Boosting Machines—GBMs) have been widely used for streamflow prediction (Kumar et al., 2023). These models are applied at various timescales, including hourly, daily, ten-daily, monthly, and seasonal forecasts, each presenting unique challenges and performance characteristics.

For this study, it was important to consider uncertainty in predictions and the forecast horizons to which the model has utility for informing the collection of water quality samples over an event. This necessitated a machine learning model that supports probabilistic regression where the goal is not only to predict the mean of the target variable but also to estimate the full predictive distribution, capturing uncertainty. Additionally, the selected model needed to accommodate both categorical and continuous variables and handle scenarios with fluctuating inflow values.

CatBoost (Prokhorenkova et al., 2018) was selected as our modelling framework for building regression models for hourly streamflow forecasting due to demonstrated capabilities in handling complex, heterogeneous data structures and its robustness in applications. CatBoost is built on the gradient boosting framework, which iteratively adds decision trees to minimise a specified loss function. Multiple comparative studies have shown that CatBoost consistently achieves high accuracy when applied to various forms of hydrological forecasting, including daily streamflow forecasting in mountainous catchments (Szczepanek, 2022), discharge estimation in compound channels (Sandilya et al., 2024), river inflow prediction (Kumar et al., 2023, 2025), monthly streamflow prediction using satellite precipitation data (Mehraein et al., 2022), river flow simulation (Rathnayake et al., 2023), and daily rainfall-runoff modelling (Chandran & Chithra, 2025). These studies demonstrate that CatBoost achieves lower mean absolute error (MAE), root mean square error (RMSE), and higher R-squared (R^2^) values, often outperforming or matching other leading GBM algorithms such as XGBoost (Chen & Guestrin, 2016) and LightGBM (Ke et al., 2017), especially in handling both categorical and continuous variables and in scenarios with moderate to high inflow values.

CatBoost supports probabilistic regression by optimising loss functions that account for both prediction accuracy and uncertainty, such as the RMSEwithuncertainty loss (Duan et al., 2020; Malinin et al., 2021). This loss function extends the traditional RMSE by incorporating terms that penalise both the mean prediction error and the misestimation of predictive uncertainty, enabling the model to output distributional forecasts rather than point estimates. Other core algorithmic advances in CatBoost include ordered boosting, which prevents target leakage and prediction shift, and ordered target statistics for categorical features, ensuring unbiased and stable model training. These innovations are particularly beneficial in time series contexts, where data leakage and overfitting are common challenges (Hancock & Khoshgoftaar, 2020; Prokhorenkova et al., 2018).

### Data preparation and feature engineering

The input dataset consisted of hourly hydrological (river height and discharge) and meteorological (rainfall) observations collected across multiple monitoring sites within the catchment. Hydrological and meteorological data were sourced from the Queensland Government’s Water Monitoring Information Portal, which provides publicly available records of water and climate observations across the state (State of Queensland Government, 2024). The data were loaded and arranged chronologically to preserve the temporal sequence required for forecasting (Hyndman & Athanasopoulos, 2018). To define the forecast objective, the target variable was created by applying a forward shift (‘lead’) to the river height time series at the target site (Tully River at Euramo). This operation aligned each set of predictor values with the river height at a future time step, enabling the model to learn and forecast river conditions at fixed forecast horizons ranging from one to 48 h ahead.

To construct the input features, we generated time-lagged versions of key hydrological predictors to capture short-term memory and flow dynamics. Lagged features were created for rainfall, river discharge, river height, and the rate of change in river height, using data from the target site on the Tully River, two upstream locations, and one adjacent site that, during major events, merges with the Tully River to form a single inundated floodplain (Fig. 2). Each variable was lagged by one to 48 h, providing the model with a comprehensive view of upstream and local conditions influencing future river height.

The final set of predictor features was grouped into categories designed to provide spatial and temporal context:

- Target feature (*n* = 1): river height at Tully River at Euramo, offset by the forecast horizon to represent river height at future time* t* + *h,* where *t* is the current time step and *h* is the forecast horizon
- Lagged features (*n* = 479): time-lagged values (1 to 48 h) of discharge, river height, and rainfall at target site (Tully River at Euramo, 113006 A), upstream sites (Tully River at Tully Gorge National Park, 113015 A; Cochable Creek at Powerline, 113004 A), and adjacent site (Murray River at Upper Murray, 114001 A)
- Temporal context features (*n* = 3): including hour, day, and month
- Discharge features (*n* = 3): flow rates for target site (Tully River at Euramo) and upstream sites (Tully River at Tully Gorge National Park and Cochable Creek at Powerline)
- Rainfall features (*n* = 2): observed hourly rainfall totals at target (Tully River at Euramo) and adjacent site (Murray River at Upper Murray)
- River height features (*n* = 6): river height and the rate of change in river height at target site (Tully River at Euramo) and upstream sites (Tully River at Tully Gorge National Park, Cochable Creek at Powerline)

The complete dataset comprised 126,986 hourly observations with 494 predictor features, partitioned into training (76,191 observations), validation (25,397 observations), and test (25,398 observations) sets as detailed in Table 1.

**Table 1:** Temporal data partitions used for model training, validation, and testing across hourly and daily forecast horizons

Forecast horizon	Training period	Validation period	Test period
Hourly (1 h, 2 h, 3 h, 4 h, 5 h, 6 h, and 12 h)	2008-11-03 01:00 2018-04-15 00:00	2018-04-15 01:00 2021-06-08 00:00	2021-06-08 01:00 2024-08-01 00:00
Daily (24 h and 48 h)			

### Data partitioning

#### Model development

The dataset was arranged by time and partitioned into training, validation, and testing subsets, consistent with best practice for time series modelling, where random splitting would disrupt temporal dependence (Hyndman & Athanasopoulos, 2018). The first 80% of the time series was allocated to model development, with 75% of that portion used for training and the remaining 25% for validation (i.e. 60% training, 20% validation). The final 20% of the dataset was held out as a test set for post-optimisation evaluation. Data were split consistently across all forecast horizons, with forecast targets generated using a forward shift of the river height variable at the Tully River at Euramo. The start and end times of each partition are detailed in Table 1.

#### Flow regime classification

Flow regimes were classified based on the time-weighted flow duration curve, derived from daily instantaneous observations collected between 1972 and 2025, assuming a fixed height–discharge relationship over the study period. The flow duration curve represents the proportion of time that a given discharge is equalled or exceeded. In this paper, flows are expressed as percentiles, which are complementary to exceedance probabilities (e.g. a 5% exceedance corresponds to the 95th percentile). Four flow regimes were defined as follows: low flow (≤ 20th percentile; ≤ 1.9 m), moderate flow (> 20th to ≤ 80th percentile; 1.9–3.2 m), high flow (> 80th to ≤ 95th percentile; 3.2–5.0 m), and extreme flow (> 95th percentile; > 5.0 m).

### Bayesian optimisation of hyperparameters

The optimisation process was completed using the rBayesianOptimization package (Yan, 2024), which applies Gaussian process regression to iteratively evaluate the hyperparameter configurations. The optimisation was performed separately for each forecast horizon model, allowing horizon-specific parameter tuning. The best performing hyperparameter set for each horizon was then used for final model training and validation. For each forecast horizon, a training and validation dataset was created using the selected feature set and the corresponding target variable (i.e. river height at 1- to 6-h, 12-h, 24-h, and 48-h horizons). These datasets were formatted using catboost.load_pool() to produce training and validation pools. The final model was trained using catboost.train(), which fits the model on the training pool while monitoring performance on the validation pool. This allowed the model to benefit from early stopping to avoid modelling overfitting to ensure that it was generalisable to unseen data.

### Model performance evaluation

Model performance was evaluated across the entire test series and separately for observations above the 95th percentile of river height (extreme flow regime). For each forecast horizon, the final CatBoost model was trained on the training data with the optimal hyperparameters and evaluated on a held-out test dataset. The test data were excluded from both training and hyperparameter optimisation, providing an unbiased assessment of forecast accuracy. Using the following equations, we evaluated the model through four different contexts: accuracy, bias, variance explanation, and uncertainty reliability. Here, ${y}_{i}$ is the observed value at the ${i}^{th}$ time step, $\widehat{{y}_{i}}$ is the predicted ${i}^{th}$ data point, $\overline{y }$ is the mean of the observed values, n is the number of observations.

#### Absolute error magnitude

Root mean square error (RMSE) measures the average magnitude of prediction errors as the square root of the mean squared difference between predicted and observed values. It uses the following formula (Moriasi et al., 2015):

$$\mathrm{R}\mathrm{M}\mathrm{S}\mathrm{E}=\sqrt{\frac{1}{n}\sum\limits_{i=1}^{n}{\left({y}_{i}-\widehat{{y}_{i}}\right)}^{2}}$$

#### Variance explanation and predictive efficiency

The coefficient of determination (R^2^) assesses the strength of the linear association between observed and predicted values. In this study, we define R^2^ as the squared Pearson correlation between observations and predictions. Values range between 0 and 1, with larger values indicating a stronger linear association between observed and predicted values (Moriasi et al., 2015). It is calculated using the following formula:

$${\mathrm{R}}^{2}={\left[\frac{\sum\limits_{i=1}^{n}\left({y}_{i}-\overline{y }\right)\left(\widehat{{y}_{i}}-\overline{\widehat{y} }\right)}{\sqrt{\sum\limits_{i=1}^{n}{\left({y}_{i}-\overline{y }\right)}^{2}\sqrt{\sum\limits_{i=1}^{n}{\left(\widehat{{y}_{i}}-\overline{\widehat{y} }\right)}^{2}}}}\right]}^{2}$$

The Nash–Sutcliffe efficiency (NSE) measures model skill relative to the mean of the observed data. It quantifies how well predictions reproduce the variability of observations. An NSE of 1 indicates perfect agreement, values between 0 and 1 reflect performance better than the mean benchmark, and values below 0 indicate poorer performance (Moriasi et al., 2015). It uses the following formula:

$$NSE=1-\frac{\sum\limits_{i=1}^{n}{\left({y}_{i}-\widehat{{y}_{i}}\right)}^{2}}{\sum\limits_{i=1}^{n}{\left({y}_{i}-\overline{y }\right)}^{2}}$$

#### Bias and relative error measures

Metrics in this category evaluate whether the model systematically overestimates or underestimates observed values, and whether error magnitudes are meaningful relative to the natural variability of the data. They provide insight into directional bias, proportional error, and the comparability of performance across different scales. Mean error (ME) measures the average signed difference between observed and predicted values. Positive ME indicates underpredictions, and negative ME indicates overpredictions (Moriasi et al., 2015). It uses the following formula:

$$ME=\frac{1}{n}\sum\limits_{i=1}^{n}\left({y}_{i}-\widehat{{y}_{i}}\right)$$

Percent bias (PBIAS) quantifies the average tendency of predictions to deviate from observations, expressed as a percentage of the total observed sum. Positive values indicate underprediction, while negative values indicate overprediction (Moriasi et al., 2015). It uses the following formula:

$$PBIAS=100\times \frac{{\sum}_{i=1}^{n}\left({y}_{i}-\widehat{{y}_{i}}\right)}{{\sum}_{i=1}^{n}{y}_{i}}$$

The ratio of RMSE to the standard deviation of observations (RSR) standardises RMSE by dividing it by the variability in the observed data. This measure facilitates comparison of model performance across different sites or variables with differing scales (Moriasi et al., 2015). It uses the following formula:

$$RSR=\frac{\sqrt{\frac{1}{n}\sum\limits_{i=1}^{n}{\left({y}_{i}-\widehat{{y}_{i}}\right)}^{2}}}{\sqrt{\frac{1}{n-1}\sum\limits_{i=1}^{n}{\left({y}_{i}-\overline{y }\right)}^{2}}}$$

### Uncertainty quantification and interval reliability

These metrics assess both the coverage of observed values within the estimated intervals and the relative sharpness of those intervals compared to the variability in the observed data. Together, they provide insight into whether the uncertainty estimates are both reliable and informative. The P-factor quantifies the proportion of observed values that fall within the model’s prediction interval (Yang et al., 2008). Values close to 1 indicate that most observations are captured by the interval, reflecting high coverage. It uses the following formula:

$$P-factor=\frac{1}{n}\sum\limits_{i=1}^{n}I\left[{CI}_{i}^{low}\le {y}_{i}\le {CI}_{i}^{high}\right]$$

The *R*-factor measures the average width of the prediction interval relative to the standard deviation of observed values (Yang et al., 2008). Lower values indicate sharper intervals, while higher values reflect greater uncertainty. It uses the following formula (Yang et al., 2008):

$$R-factor=\frac{\frac{1}{n}\sum\limits_{i=1}^{n}\left({CI}_{i}^{high}-{CI}_{i}^{low}\right)}{\sqrt{\frac{1}{n-1}\sum\limits_{i=1}^{n}{\left({y}_{i}-\overline{y }\right)}^{2}}}$$

## Results and discussion

### Model evaluation

**Fig. 3:**
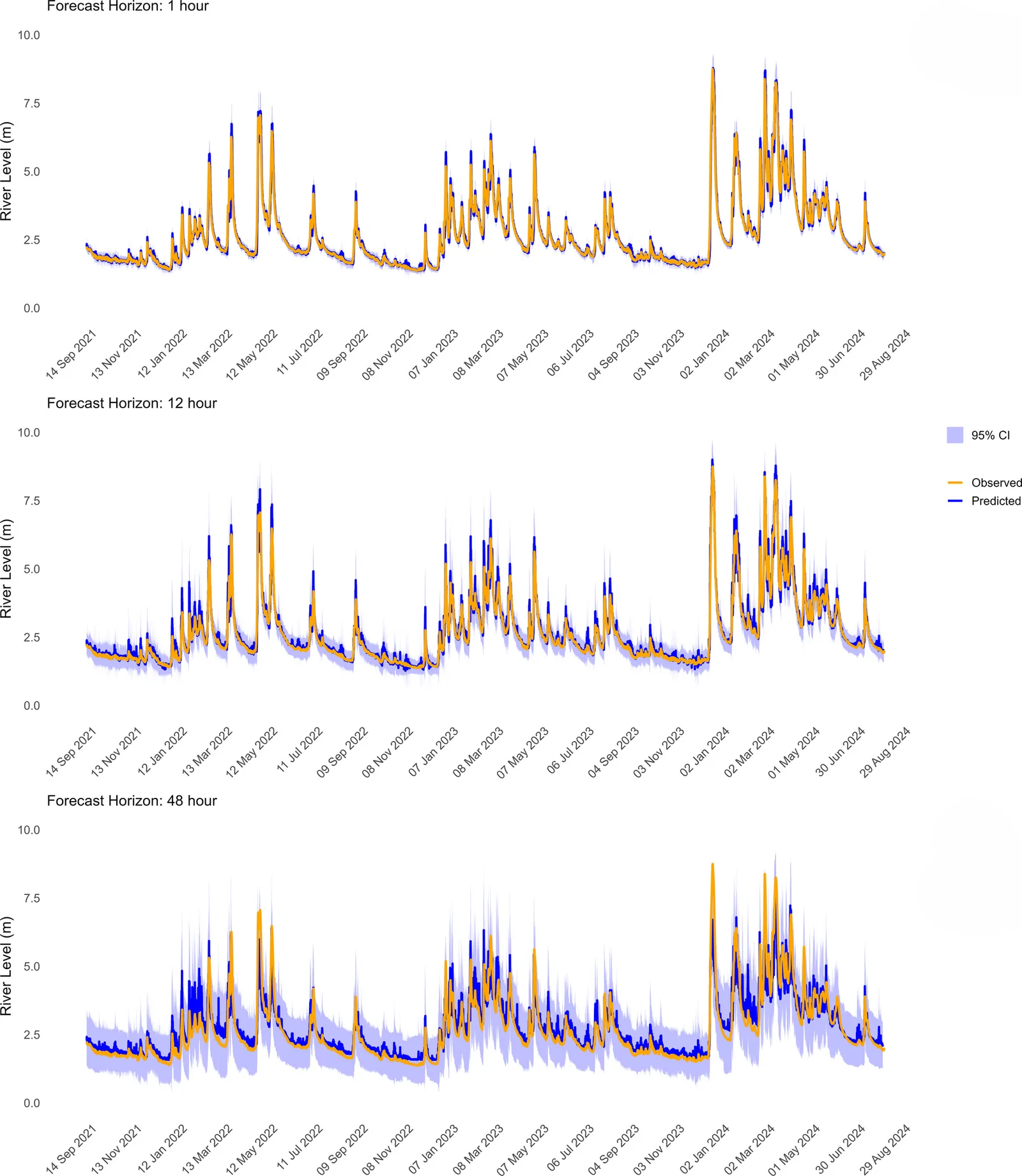
Observed and predicted river heights for 1-h, 12-h, and 48-h forecast horizons on a section of the test dataset. Blue lines represent observed river heights, while semi-transparent orange lines show predicted values

This study applied machine learning to river forecasting in a tropical catchment, demonstrating predictive capabilities that can adaptively inform the design and timing of water quality sampling programs. We found that predictive performance was closely linked to the length of the forecast horizon, with short horizon models (1 to 6 h) exhibiting stronger accuracy and reliability compared to long horizon models (12, 24, and 48 h). The performance of long-range forecast models (e.g. 12 h, 24 h, and 48 h) declined with increasing lead time, with both under- and overpredictions observed across the test period. The visual comparison between observed and predicted hydrographs for the 1-h, 12-h, and 48-h horizons (Fig. 3) illustrates these patterns. For 1-h horizons, predictions closely matched observations, capturing both the magnitude and timing of peaks with minimal deviation. For 12-h horizons, the model largely followed the hydrograph shape and timing but showed more frequent deviations, particularly on sharp rising limbs. By the 48-h horizon, errors became more evident, with peaks occasionally smoothed and event timing occasionally misaligned; however, the model still reproduced the overall hydrograph shape and timing close enough to provide meaningful guidance for sampling logistics. These trends highlight the trade-off between lead time and accuracy, while also demonstrating the operational utility of long horizon forecasts, particularly if systematic tendencies such as underprediction are recognised and can be incorporated into planning.

We evaluated model performance across four contexts: absolute error magnitude, variance explanation and predictive efficiency, bias and relative error measures, and uncertainty quantification and interval reliability (Table 2). Evaluating metrics across these categories allowed us to assess both model performance and their feasibility for supporting near real-time sampling during hydrological events in a tropical system. RMSE values provide a direct measure of how far forecasts deviate from observed river heights, allowing us to evaluate the absolute error magnitude across the different model forecast horizons. Across short horizon forecasts (1 to 6 h) the RMSE was below 0.06 m (m), suggesting that the predicted river height is within a few centimetres of the observed height value. From an operational perspective, this level of accuracy provides confidence in short horizon forecasts to trigger automated samplers near the true event timing. This can support effective sampling during periods when human operators are unavailable to monitor or manually activate equipment. At longer forecast horizons, RMSE increased to 0.16 m at the 12-h horizon, 0.35 m at the 24-h horizon, and 0.60 m at the 48-h horizon. These larger deviations suggest that in longer-range forecasts, particularly at daily scales, the models may miss the precise height of flow peaks or underestimate their duration. This increases the risk that sampling events are mistimed and fail to capture critical phases of an event, such as the rising limb or peak flow.

**Table 2:**
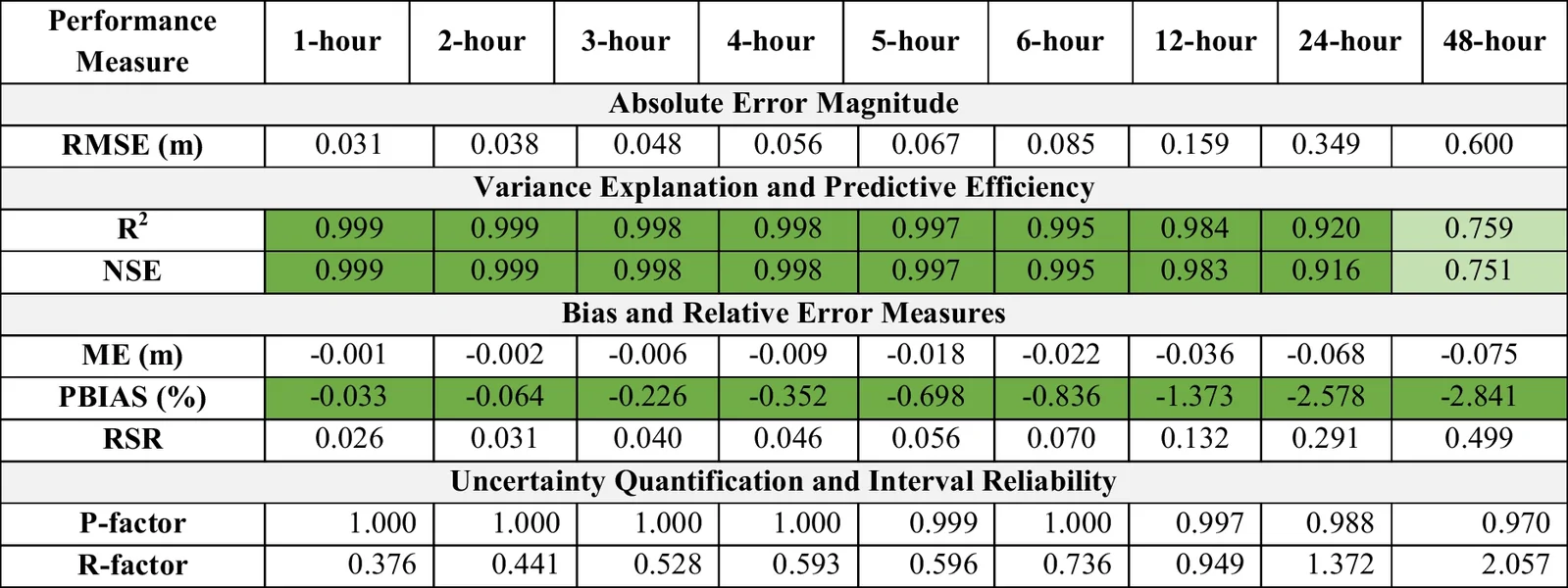
Test-set performance across forecast horizons, summarised by categories of evaluation metrics: absolute error magnitude (RMSE, metres (m)); variance explanation and efficiency (R^2^, NSE); bias and relative error (ME, metres (m); PBIAS, %; RSR); and uncertainty and interval reliability (P-factor, 95% CI coverage; R-factor, normalised CI width). Colour coding indicates model performance ratings based on the evaluation criteria of Moriasi et al. (2015) for R^2^, NSE, and PBIAS only. Darker green cells represent statistics rated as very good, while lighter green cells represent statistics rated as good. Note that these evaluation criteria were developed for daily flow, which is the finest temporal resolution assessed in Moriasi et al. (2015). The remaining metrics (RMSE, ME, RSR, P-factor, R-factor) do not have established performance thresholds in that framework and were therefore not colour coded

Forecast performance metrics such as the coefficient of determination (R^2^) and Nash–Sutcliffe efficiency (NSE) quantify how well the model reproduces the variability and structure of the observed data, indicating its ability to capture and predict hydrological dynamics. For short horizon forecasts, R^2^ and NSE values ranged from 0.995 to 0.999, showing near-perfect alignment between predicted and observed variability and confirming the models’ strong ability to capture hydrograph dynamics at fine temporal scales (Table 2). These results demonstrate that short horizon forecasts can inform the scheduling of sampling to capture the full hydrograph shape and timing, including the rising limb, peak, and falling limb. Performance declined at longer horizons, with R^2^ dropping to 0.984 at the 12-h horizon, 0.920 at the 24-h horizon, and 0.759 at the 48-h horizon; NSE values followed a similar trend (0.983, 0.916, and 0.751, respectively). This suggests that while long horizon forecasts were able to reproduce overall flow patterns, their ability to represent event details reduced progressively as the forecast horizon increased.

Bias metrics quantify the systematic tendency of model forecasts to either overpredict or underpredict river height. The mean error (ME) represents this bias on an absolute scale (metres), whereas the percent bias (PBIAS) expresses it relative to observed river heights. Across all forecast horizons, ME values were consistently negative, ranging from −0.001 m at 1-h to −0.075 m at the 48-h horizon (Table 2). These results indicate a slight overprediction bias but of negligible magnitude in practical hydrological terms; further supported by PBIAS results, which show relative overprediction across all horizons (−0.03% to −2.84%) (Table 2). Visual inspection of individual events in Fig. 3 confirms this pattern, showing that the overprediction tendency is most apparent during flood peaks at shorter forecast horizons (1 h and 12 h), while predictions more closely match observations during baseflow and recession periods. For the specific application of timing water quality sample collection to capture hydrograph phases (rising limb, peak, falling limb), this slight overprediction bias does not diminish the model’s utility, as the critical information is the temporal structure and timing of the hydrograph rather than absolute peak magnitude.

Different from the previously discussed metrics, which are retrospective statistics measuring model skill against observed data, the P-factor and R-factor are forward-looking metrics that quantify, respectively, the likelihood that the observed river height will fall within the prediction interval and the width of that interval relative to the natural variability of the system. Across all models, P-factor values were equal to or greater than 97%, indicating highly reliable uncertainty bounds and showing that the intervals adequately capture observed variability (Table 2). Similar to the other performance metrics, the R-factor followed the same pattern across horizons, demonstrating that prediction intervals were narrower and more precise at short horizon forecasts (1 h = 0.376) and became progressively wider at longer horizons (48 h = 2.057). Together, the P-factor and R-factor provide operational confidence by showing not only that the hydrograph shape and timing will be captured, but also how much trust can be placed in the forecasts at different horizons if used when setting triggers for adaptive sampling.

**Fig. 4:**
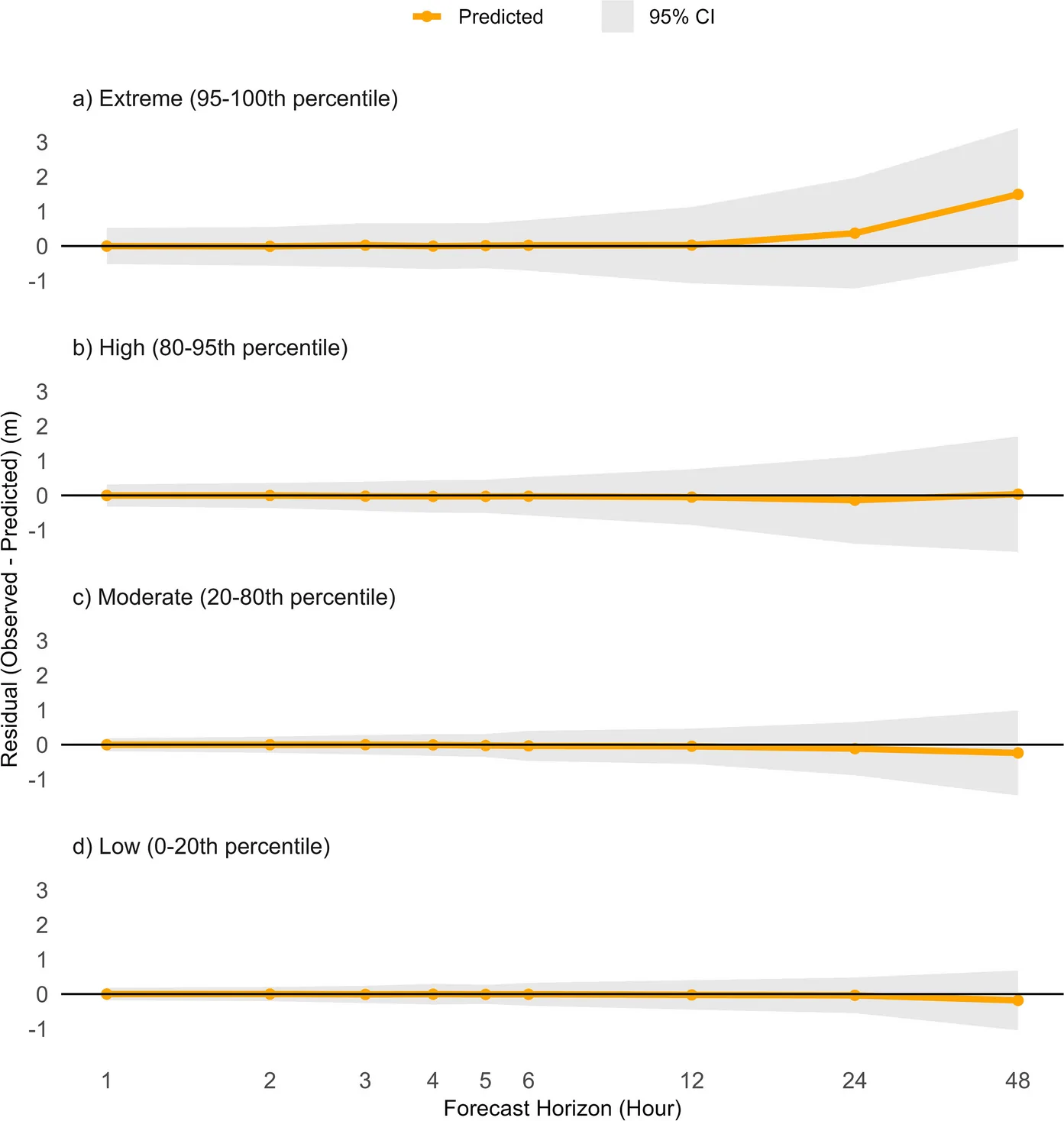
Mean residual (orange line) for each flow regime at each forecast horizon during the entire test period. Flow regimes are (**a**) extreme flow (> 95th percentile; > 5.0 m), (**b**) high flow (> 80th to ≤ 95th percentile; 3.2–5.0 m), (**c**) moderate flow (> 20th to ≤ 80th percentile; 1.9–3.2 m), and (**d**) low flow (≤ 20th percentile; ≤ 1.9 m). Shaded bands represent the mean 95% confidence intervals derived from the model-estimated predictive variance, and the black line denotes the zero-residual reference

**Fig. 5:**
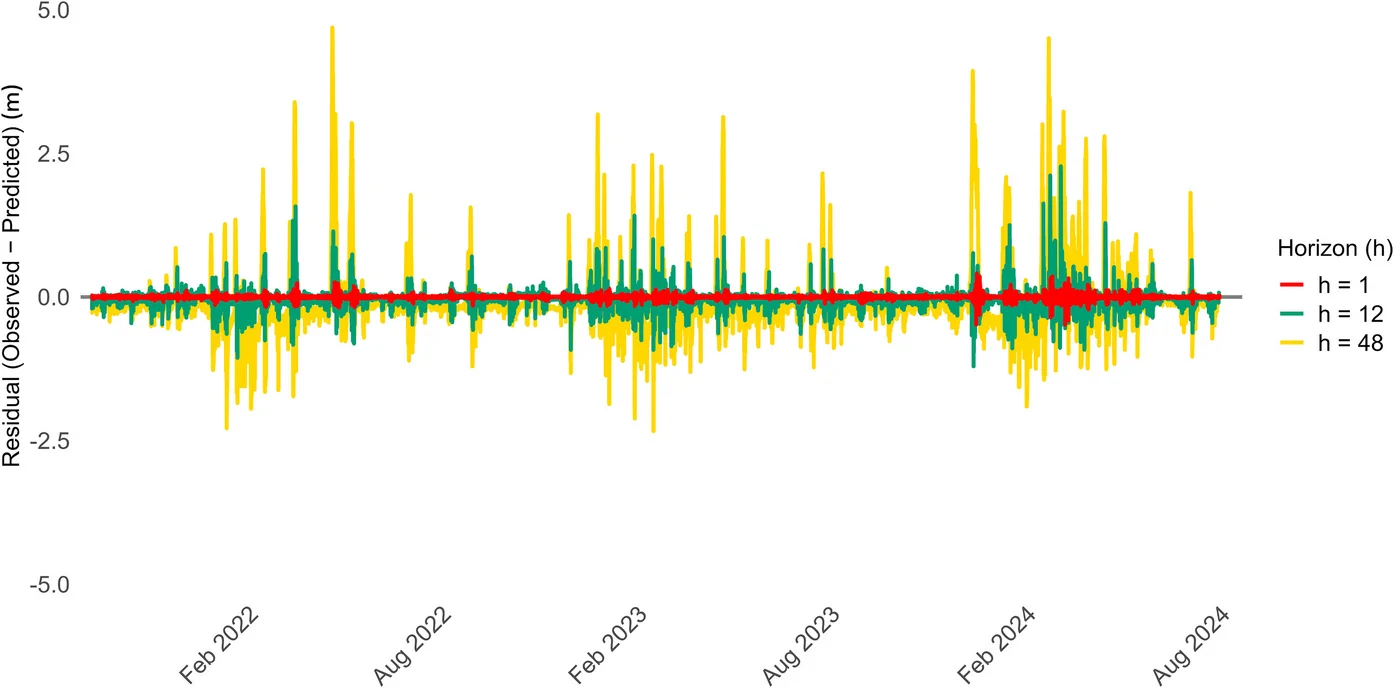
Residuals (Observed − Predicted) for three forecast horizons across the test dataset. Coloured lines show residuals at 1-h (red), 12-h (green), and 48-h (yellow) forecast horizons. Values above zero indicate underprediction; values below zero indicate overprediction

Figure 4 illustrates how forecast performance and uncertainty vary across different flow regimes. Forecasts are shown for four flow regimes: extreme flow (> 95th percentile; > 5.0 m river height), high flow (> 80th to ≤ 95th percentile; 3.2–5.0 m), moderate flow (> 20th to ≤ 80th percentile; 1.9–3.2 m), and low flow (≤ 20th percentile; ≤ 1.9 m). Across all flow regimes, forecast uncertainty increases with horizon length, as indicated by the widening of the 95% confidence intervals. Divergence between observed and predicted river heights becomes more pronounced from 24 to 48-h horizons under extreme flow conditions (> 5 m river height). This divergence indicates bias, where the mean predicted value is up to 1.5 m lower than the observed river height. Forecasts for low, moderate, and high flow remained more stable, with residuals close to zero even at longer horizons. These results suggest that while uncertainty and bias are greatest during extreme flow, forecasts across a range of flow regimes still reproduce the general hydrograph pattern and provide useful guidance for planning targeted water quality sampling. Residual analysis in Fig. 5 further demonstrates model performance across different forecast horizons. At short horizons, residuals are narrowly distributed around zero, indicating low bias and variance. At longer horizons (12, 24, and 48 h), residuals become more dispersed, with occasional large positive deviations during extreme flow conditions. Because residuals are defined as Observed − Predicted, positive values indicate that the model underpredicted river height, meaning predicted values were lower than those observed. These positive spikes reflect a tendency for the model to underestimate river height at event peaks. This pattern is consistent with the increasing uncertainty and widening predictive intervals shown in Fig. 4, where the spread of residuals grows with forecast horizon, particularly under high and extreme flow regimes.

### Predicting extreme flow events

Short horizon forecasts closely reproduced the hydrograph shape during extreme flow events, capturing both the rising and falling limbs with high accuracy (Fig. 3). Rapid rises in river height were closely tracked, with RMSE values below 0.20 m, variance explanation exceeding 0.96 (R^2^, NSE), and minimal relative error (RSR ≤ 0.19; Table 3). Bias was negligible (ME near zero; PBIAS < 0.5%), indicating that peaks and subsequent recessions were captured without systematic over- or underprediction. Prediction intervals were also reliable, with P-factor values between 0.993 and 1.000 and narrow R-factors (~1.1–1.5), showing that both the steepness of the rising limb and the trajectory of the falling limb were consistently within the model’s uncertainty bounds. The predictive accuracy demonstrated by short horizon forecast models during extreme flow events suggests that their implementation into water quality monitoring regimes would provide a reliable basis for triggering event-based sampling, helping to ensure that key hydrological moments driving pollutant transport are captured.

**Table 3:**
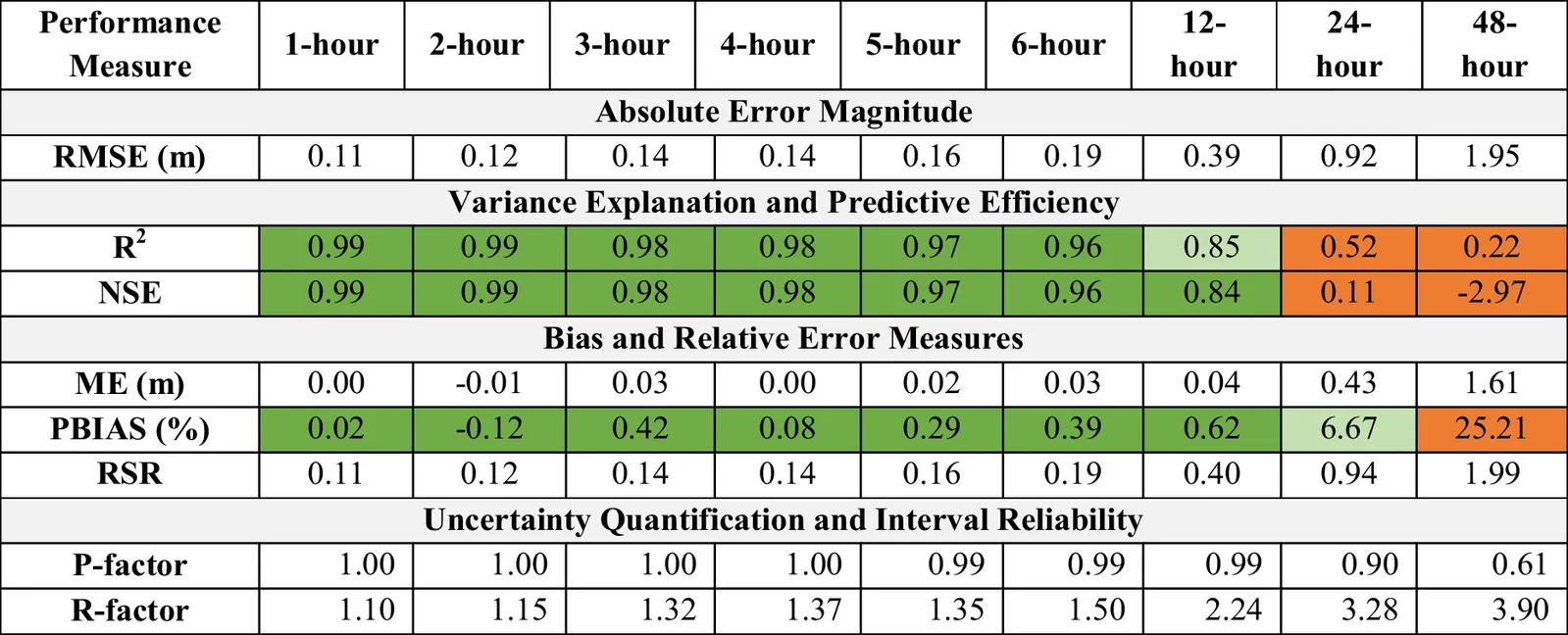
Test-set performance across forecast horizons for extreme flow conditions (observed river height ≥ 95th percentile): absolute error magnitude: RMSE (metres); variance explanation and efficiency: R^2^, NSE; bias and relative error: ME (metres), PBIAS (%), RSR; uncertainty and interval reliability: P-factor (95% CI coverage) and R-factor (normalised CI width). Colour coding indicates model performance ratings based on the evaluation criteria of Moriasi et al. (2015) for R^2^, NSE, and PBIAS only. Dark green cells represent statistics rated as very good, lighter green cells represent statistics rated as good, and orange cells represent statistics rated as not satisfactory. Note that these evaluation criteria were developed for daily flow, which is the finest temporal resolution assessed in Moriasi et al. (2015). The remaining metrics (RMSE, ME, RSR, P-factor, R-factor) do not have established performance thresholds in that framework and were therefore not colour coded

By the 12-h horizon, the model retained skill in predicting the overall rise and fall of the hydrograph during extreme flow events, but with a diminished response. RMSE increased (0.39 m), R^2^ dropped to 0.85, and relative error rose (RSR = 0.40), suggesting a tendency to smooth over sharp transitions. The rising limb was still broadly captured; however, peaks were less sharply defined, and the falling limb became less precise. While uncertainty widened (R-factor = 2.25), prediction intervals still included nearly all observed values (P-factor = 0.99), indicating that the main shape and timing of the hydrograph remained intact but with reduced sharpness and growing timing errors. These results indicate that 12-h forecasts during extreme flow events remain operationally valuable, as water quality sampling strategies rely more on detecting shifts in flow magnitude and identifying rising and falling limbs than on precise river heights. Therefore, even with some loss of accuracy in absolute water-level estimates, the forecasts provide a reliable basis for anticipating hydrological changes that drive event-based water quality sampling.

At longer horizons, from 24 to 48 h, the models struggled to replicate the hydrograph shape and timing of extreme flow events. Forecasts tended to sustain higher river heights for longer, leading to systematic overprediction (ME = 0.43–1.61 m; PBIAS up to 25%). This produced hydrographs that overstated both the magnitude and duration of peaks while failing to accurately capture the falling limb. Model efficiency deteriorated at the 48-h horizon, with negative R^2^ and NSE values, indicating that forecasts at this lead time added no predictive value beyond the mean flow. Error magnitudes were large (RMSE > 1.8 m), and prediction intervals widened substantially (R-factor ≈ 3.9), with failure to reliably capture observed extreme flow events (P-factor = 0.61). These results indicate that forecasts from 24- to 48-h horizons lack the accuracy and reliability needed to anticipate river height changes during extreme flow events, limiting their operational value for event-based water quality sampling.

### Model feature importance and transferability

**Fig. 6:**
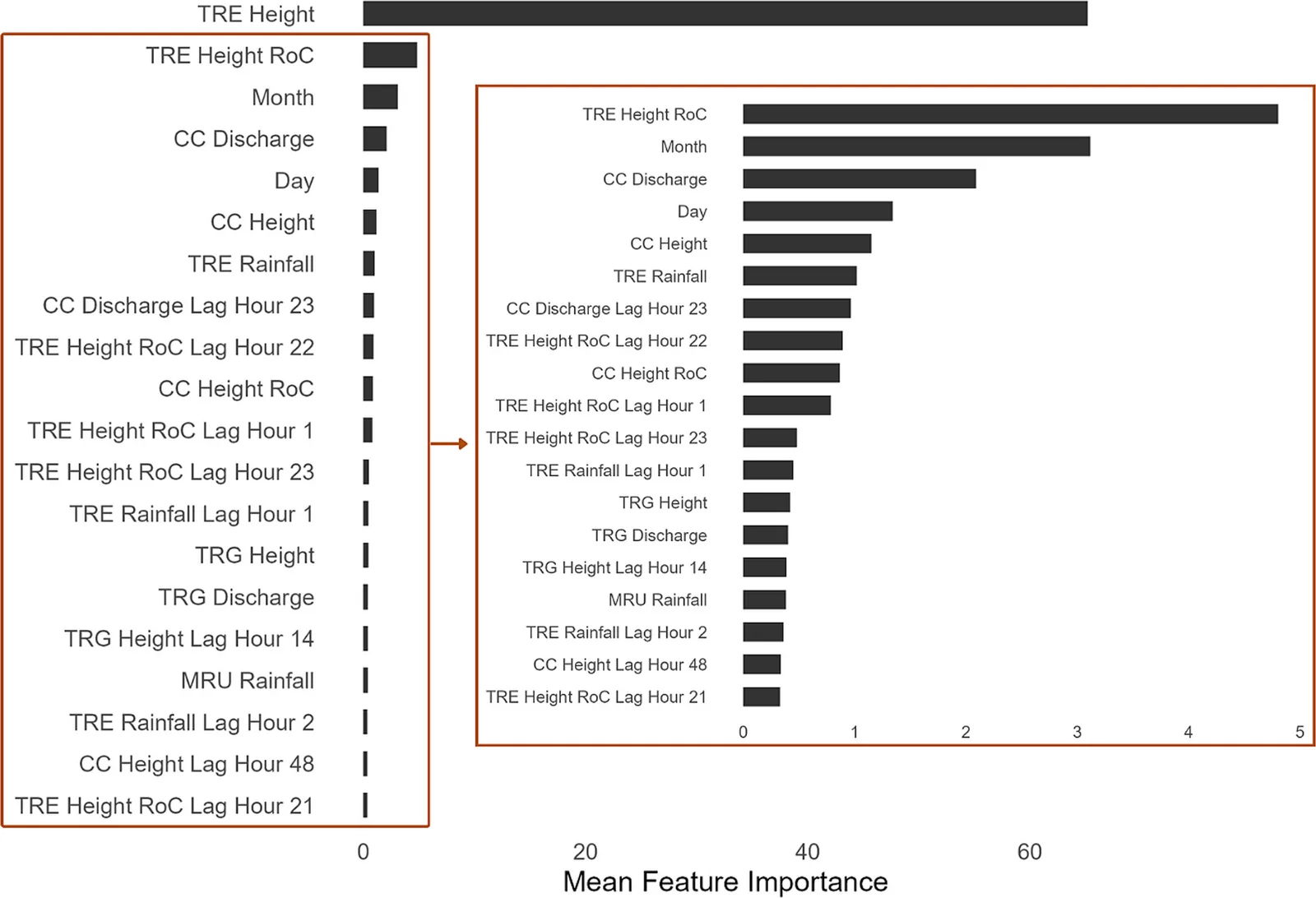
Top 20 features (out of 494) ranked by mean importance across all forecast horizons. The inset shows a magnified view of the 2nd to 20th most important features. Rate of change in river height is labelled as ‘RoC’. Site abbreviations are as follows: TRE, Tully River at Euramo (target site); CC, Cochable Creek at Powerline (upstream); TRG, Tully River at Tully Gorge National Park (upstream); MRU, Murray River at Upper Murray (adjacent site that connects with the Tully River when floodplains are inundated)

We calculated the average feature importance from CatBoost models trained independently at each forecast horizon to identify the most important predictors of river height at the target site. Figure 6 presents the top 20 features (out of 494) ranked by mean importance across all horizons. The single most influential predictor was the river height at the target site at time* t* = 0 (TRE height; 65.2%), confirming that current conditions capture the majority of information needed for forecasting. The rate of change in river height at the target site (TRE rate of change; 4.8%) was the next most important feature, although its contribution was small in comparison. Temporal context variables such as month (3.1%) and day (1.3%) also appeared among the top predictors, suggesting the model detected weak seasonal or diurnal influences. Upstream discharge at Cochable Creek (2.1%) and river height at the same site (1.2%) provided minor contributions, while Tully Gorge discharge and height each accounted for less than 0.5%. Local rainfall at TRE (1.0%) was similarly of low importance, indicating that while rainfall–runoff processes and upstream inputs play a role, their influence was minor relative to local river height at the forecast origin. Several lagged predictors, including lagged discharge, rainfall, and river height rate of change, also appeared in the top 20 features, although each accounted for less than 1% of the total importance. Their presence suggests they may provide supplementary information on antecedent conditions, particularly at longer forecast horizons.

The importance of river height at the target site at time *t* = 0 (65.2%) in our study is consistent with findings from other gradient boosting decision tree regression applications to short-term hydrological forecasting, though direct comparisons are limited by differences in target variables and forecast horizons. Szczepanek (2022) reported that upstream discharge at* t* = 0 contributed approximately 80% of feature importance in daily streamflow forecasting using XGBoost. Similarly, our study identified lagged features (rainfall, discharge, and rate of change in river height) among the top 20 most important features, which aligns with the findings of Chandran and Chithra (2025), who found that lagged discharge accounted for 75.6% of feature importance in daily rainfall–runoff modelling using CatBoost. While these studies forecasted discharge (streamflow) and our study forecasts river height, all studies demonstrate the autoregressive nature of hydrological systems at hourly to daily timescales, where a single feature (current or recent flow/height at the target or upstream location) overwhelmingly dominates (65–80%). The relative importance of secondary predictors, however, varied considerably across studies. Rainfall accounted for 14% of importance in Chandran and Chithra (2025) but only 1% in our analysis, which may reflect both the difference between forecasting discharge versus river height, and localised catchment hydrology.

The models were trained and optimised using the available data and local hydrology. They show that the most important predictor of river height at the target site is the river height 1 h prior. It is important to consider that systems with different hydrological characteristics may yield different features of importance. As outlined in Lopez et al. (2025), the Tully River catchment is small in area (relative to other catchments in the similar geographic region), wet, and highly responsive, with high runoff efficiency (80 ± 15%), meaning that the local river height gauge at *t* = 0 captures much of the information needed for short- to medium-range forecasts. In contrast, another northern Australian catchment, the Burdekin, has low-runoff efficiency (9 ± 9%) and much higher inter- and intra-annual variability. The Burdekin catchment also receives substantial inflows from multiple upstream sub-catchments, with high-flow events taking days to weeks to travel to the lower river. In larger catchments, such as the Burdekin in Queensland, Australia, river height at *t* = 0 is less likely to dominate model predictions, and upstream discharge and lagged discharge variables would be expected to have far greater importance. This was not explored in the present study, and further research is required to compare feature importance profiles between large, low-runoff catchments and lower order, fast-responding river systems such as the Tully.

### Limitations

This study adopts a direct multi-horizon forecasting strategy, in which separate CatBoost models are trained for each forecast lead time (from 1- to 48-h horizons), avoiding the error propagation typical of recursive approaches that feed a model’s own predictions back as inputs (e.g. Cheng et al., 2020). These recursive methods are often coupled with continual validation and adaptive learning, allowing the model to update its internal parameters based on recent performance. In practice, the model developed here generates predictions, compares them against observed values, and adjusts accordingly, creating a looped workflow that can adapt to evolving hydrological conditions in near real time. Model performance was evaluated using a fixed train/validation/test split, which provides a straightforward benchmark. However, research indicates that static splits may not adequately capture the temporal dependencies and evolving dynamics characteristic of hydrological systems (Shen et al., 2022). Furthermore, longer training datasets are particularly important for improving the skill and robustness of hydrological forecasting models (Fathi et al., 2025). Walk-forward or time series cross validation techniques, which incrementally expand the training window and validate on future observations, provide a more realistic assessment of operational performance and make more efficient use of available data (Ali et al., 2024; Hyndman & Athanasopoulos, 2021). Incorporating walk-forward validation into future iterations of this study could increase the model’s responsiveness to changing hydrological regimes, improve generalisability across conditions, and better support near real-time deployment in operational water quality monitoring programs.

### Practical applications for water quality monitoring program

In this study, we assume that pollutant load estimates are derived from a subset of samples, representing a wider set collected across the hydrograph or event horizon. This assumption of oversampling is acknowledged as a working premise to link forecasts with sampling strategy and to assess the relevance of our case study to broader water-monitoring programs. This framing provides the basis for exploring how our forecasting approach could potentially optimise event-based sampling by reducing the costs in resources and effort associated with oversampling, without compromising on the accuracy of pollutant load estimates.

The results of this case study demonstrate the potential to obtain a reliable picture of hydrograph shape and timing from the onset of an event. Using machine learning methods as applied in this study, low flow conditions are predicted with high accuracy across all forecast horizons. Moderate events can be reasonably predicted up to 24 h in advance, including both hydrograph shape and timing. Once an event is underway, high-flow conditions can be predicted with strong skill up to 12 h ahead. The sampling strategy implications of these findings are that sampling intervals can be increased when there is advance warning of a moderate event within 24 h. In the case of a high-flow event, the peak height may be predicted with minimal bias; however, continuous assessment throughout the event is essential due to higher variance, with confidence increasing as the high-flow period approaches. Extreme events exhibit substantially lower predictive performance, regardless of forecast horizon with higher variance relative to other flow regimes and a pronounced negative bias beyond 24 h. Consequently, the sampling strategy for extreme events at the Tully River at Euramo should aim to obtain sufficient samples near the peak while reserving capacity for sampling during the high and moderate flow recession.

**Fig. 7:**
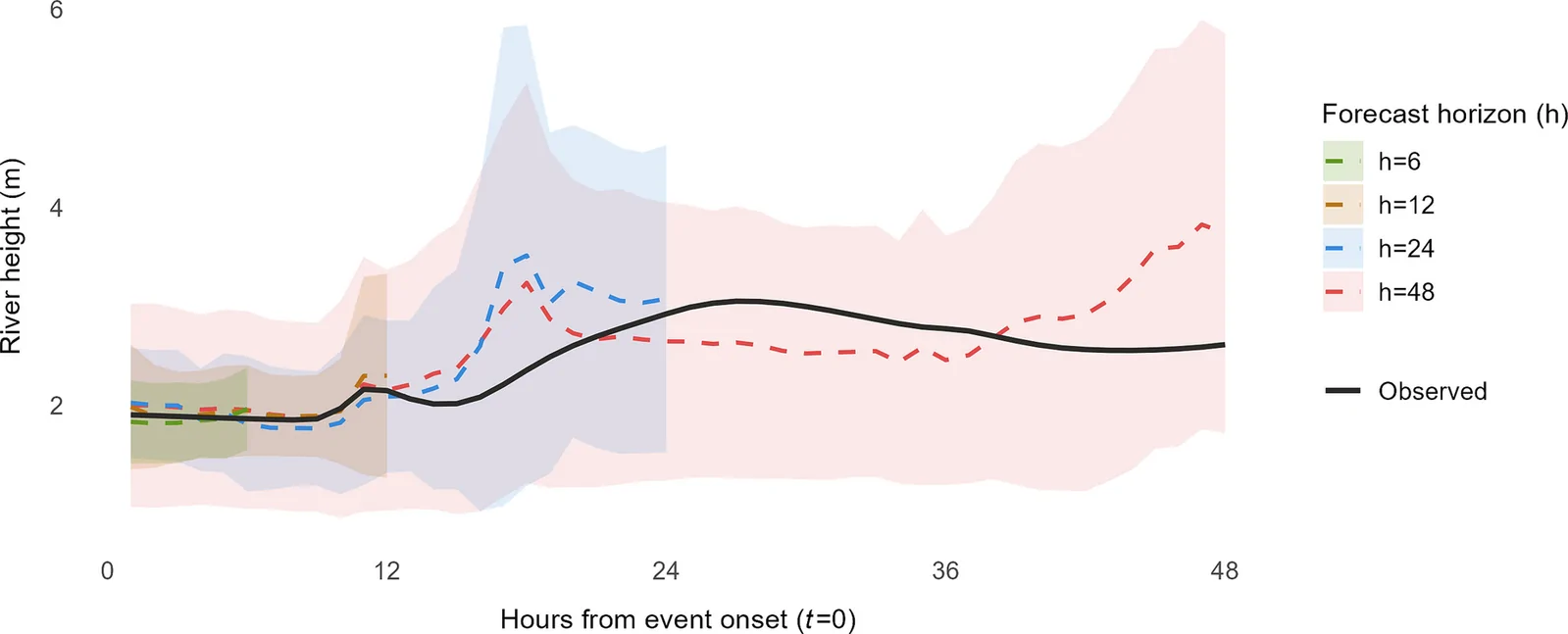
Example of information that the CatBoost models provide to samplers at the onset of an event when looking to collect water quality samples at the Tully River at Euramo. The *x*-axis starts at the onset of the event (*t* = 0) and extends through 48 h. The solid black line shows observed river height. Dashed lines show CatBoost point forecasts issued at four forecast horizons (6 h, 12 h, 24 h, and 48 h), with shaded bands indicating the corresponding 95% confidence intervals

Figure 7 illustrates how the trained CatBoost forecasts can inform sampling strategies from the onset of an event. Prior to this work, water quality monitoring teams relied on meteorological rainfall forecasts and contextual knowledge of how past events had progressed at the site. Our models add a quantitative picture of the predicted river height trajectory across multiple forecast horizons, along with the associated uncertainty bounds. In the event shown in Fig. 7, the 6-h and 12-h forecasts track the observed hydrograph closely. The 24-h and 48-h forecasts deviate from the observed trajectory but remain within the 95% confidence intervals of the forecasts. The forecasts are not intended to be exact. Their value lies in giving samplers an early indication of the type of event, predicted peak timing, and the expected magnitude. For example, the event in Fig. 7 points to a moderate event with a predicted peak beyond the 48-h forecast horizon. This is sufficient information to plan crew mobilisation, prepare autosamplers, and allocate bottle capacity across the rising limb, peak, and recession, with the expectation that the forecasts will be updated as the event progresses.

Sample preservation requirements are another important consideration. International standards such as AS/NZS 5667.1 and AS/NZS 5667.6 set maximum holding times for different analytes, meaning that samples must be collected, preserved, and analysed within a designated timeframe (Standards Australia, 1998a, b). This constraint further reduces scheduling flexibility and reinforces the value of forecasts that can anticipate event timing. The value that model forecasts can bring to operational aspects of water quality monitoring could be further enhanced by adopting a rolling ensemble approach, where successive forecasts are combined with recent observations to provide self-validating updates that reduce mid-event error propagation and keep sampling decisions aligned with the evolving hydrograph.

The deployment of machine learning models into water quality monitoring programs or automated sampling machines may be constrained by data availability, the risk of errors in real-time inputs, and the practicality of implementation in either cloud or edge (logger-based) environments. Real-time deployment would first require access to verified hourly data streams, together with mechanisms to detect and manage spikes, dropouts, or sensor failures that could otherwise compromise forecast reliability. Without access to quality-controlled real-time data, model prediction accuracy may deteriorate, reducing the operational value of forecasting for sampling applications. The sensitivity of forecast performance to unverified or raw data inputs was not investigated in this study but would be a valuable focus for future assessment, particularly in evaluating how forecast skill may degrade under conditions of data latency, noise, or limited verification.

The choice of processing environment involves several considerations and trade-offs. Edge deployment, where models run directly on on-site data loggers, reduces dependence on communication networks and ensures continued operation during outages, while also minimising latency by processing data immediately on site. However, this approach is constrained by the limited processing and storage capacity of logger hardware, which may restrict the deployment of complex or ensemble models. It may also be constrained by incomplete or missing input data, since edge systems may not have access to the full suite of input variables that the model was trained on, potentially reducing forecast accuracy. It is also limited by the greater maintenance burden of updating or patching models across distributed devices compared to a centralised cloud service. In contrast, cloud deployment provides scalable computational resources, allowing complex or ensemble models to be run efficiently and updated centrally with minimal maintenance overhead. It also facilitates integration across multiple data sources and sites, supporting coordinated forecasting at catchment scales. In future research, the deployment pathway should be evaluated to determine how best to balance reliability, computational capacity, and operational flexibility so that forecasts remain both accurate and operationally useful in real time.

## Conclusions

Effective water quality monitoring depends on the timing of sampling, because mistimed event collections can introduce substantial error into pollutant load estimates. By leveraging long-term environmental datasets, machine learning models can help anticipate hydrograph duration, shape, and key phases, providing practitioners with a tool to adaptively schedule sampling and achieve more reliable and consistent event coverage.

This study shows that machine learning models can provide skillful forecasts of river height in a tropical, fast-responding catchment. CatBoost models achieved high accuracy at short horizons (1 to 12 h), closely reproducing hydrograph magnitude and timing, with performance declining at longer horizons (24 to 48 h). Short horizon forecasts also tracked extreme events with high fidelity, whereas errors at longer horizons were concentrated around peaks and timing. Prediction intervals were reliable, with high coverage and narrow widths at short horizons, providing useful limits for operational decision-making. Integrating machine learning predictions into sampling programs may reduce the risk of mistimed collections, improve the representativeness of pollutant load estimates, and optimise the use of limited autosampler capacity. Practical constraints remain, including finite bottle numbers and analyte holding-time requirements that restrict retrieval windows, which supports the use of short horizon forecasts to target key periods of the hydrograph. Forecast horizons should be tailored to catchment characteristics, with short-term predictions most valuable in small, rapidly responding systems and longer horizons more applicable in larger or slower systems.

These findings have practical implications for water quality monitoring programs. By aligning predictions with operational needs, machine learning may offer a scalable tool to improve the efficiency and accuracy of river monitoring. Future work should assess the transferability of this approach to contrasting hydrological settings, evaluate the robustness of forecasts when using real-time unverified data streams, and explore ensemble and walk-forward validation methods. Progress in these areas will be critical to ensure that case study findings translate into dependable operational use in adaptive water quality monitoring.

## Data Availability

The hydrological and meteorological data used in this study are publicly available from the Queensland Government’s Water Monitoring Information Portal. The code used for data processing, model development, and analysis is openly available at: https://github.com/Reef-Catchments-Science-Partnership/2025_Wilson_ML-Hydrograph-prediction.
